# Supplementary material for: Complex‐centric proteome profiling by SEC‐SWATH‐MS
Source: Mol Syst Biol. 2019 Jan 14;15(1):e8438. doi: 10.15252/msb.20188438 (PMC6346213; doi:10.15252/msb.20188438)
Supplement: Supplementary file 8 — Dataset EV7 [file MSB-15-e8438-s008.zip › feature_plots_string/Q9NYV6.pdf]

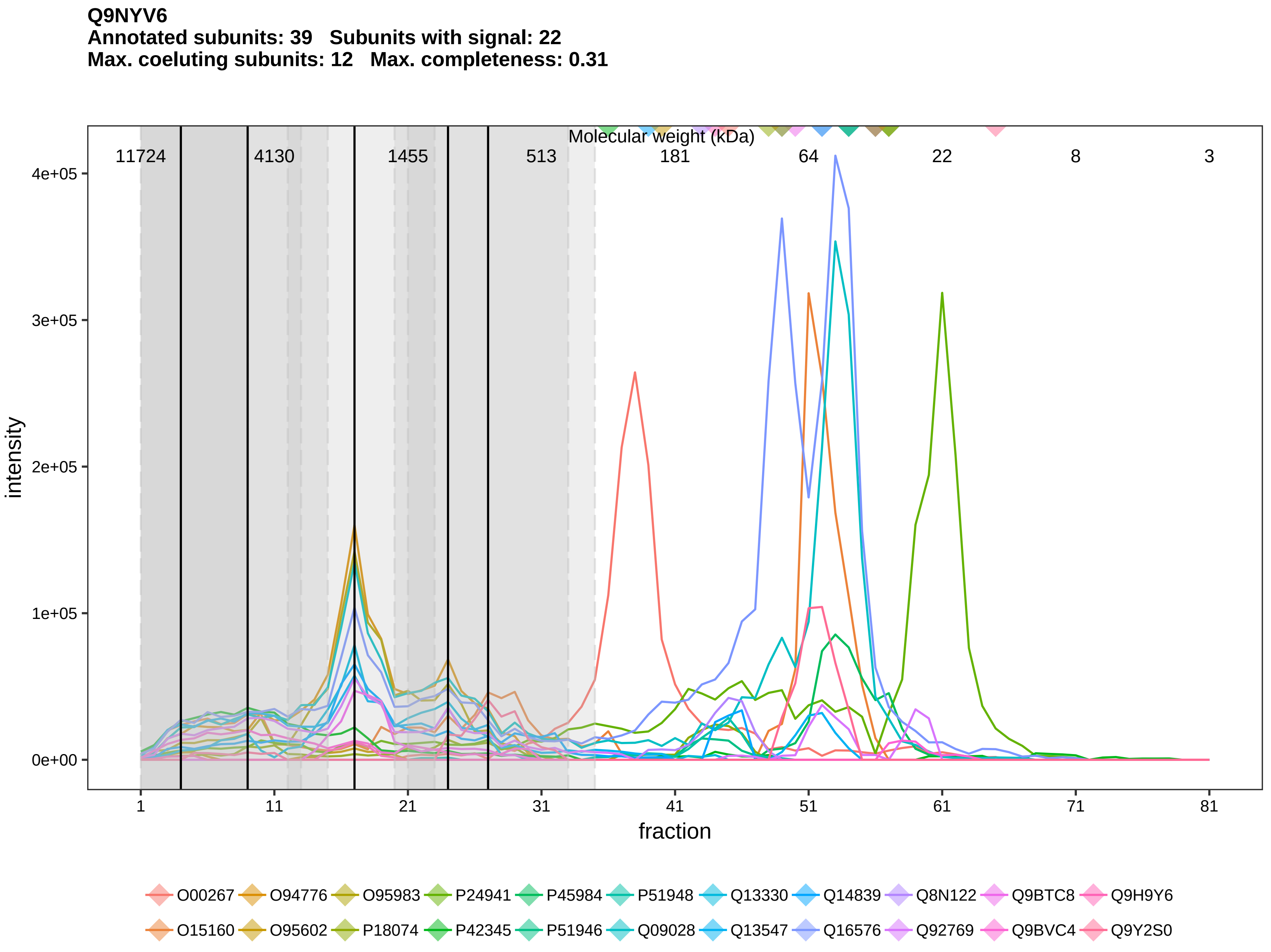

Q9NYV6
Annotated subunits: 39 Subunits with signal: 22
Max. coeluting subunits: 12 Max. completeness: 0.31
Molecular weight (kDa)
11724
4130
1455
513
181
64
22
8
3
intensity
4e+05
3e+05
2e+05
1e+05
0e+00
fraction
1
11
21
31
41
51
61
71
81
O00267
O94776
O95983
P24941
P45984
P51948
Q13330
Q14839
Q8N122
Q9BTC8
Q9H9Y6
O15160
O95602
P18074
P42345
P51946
Q09028
Q13547
Q16576
Q92769
Q9BVC4
Q9Y2S0
